# Selective Conventional Approach for Management of Bleeding Uterine Arteriovenous Malformation: A Case Report

**DOI:** 10.31729/jnma.8891

**Published:** 2025-02-28

**Authors:** Jyoti Dahal, Suman Raj Tamrakar, Rupesh Singh Basnyat

**Affiliations:** 1 Department of Obstetrics and Gynaecology, Kathmandu University School of Medicine, Dhulikhel, Kavre, Nepal

**Keywords:** *arteriovenous malformation*, *condom tamponade*, *hemorrhage*, *progesterone*

## Abstract

Uterine Arteriovenous malformation is an abnormal connection between artery and veins in the uterus. Although rare, it is an important cause of abnormal uterine bleeding in women and can lead to life threatening torrential hemorrhage. Traditionally such cases have been managed with medical therapy and hysterectomy. However, selective uterine artery embolization and hysteroscopy guided procedures are the treatment of choices. We hereby report a case of 25 years lady who presented with irregular heavy vaginal bleeding and diagnosed to have uterine arteriovenous malformation by magnetic resonance imaging. She was managed conventionally with condom tamponade during bleeding due to intrauterine procedure and later with hormonal therapy. The patient later had regular cycles avoiding hysterectomy at a young age. The objective of this case report is to share the successful application of uterine balloon tamponade (UBT) for the active management of bleeding uterine arteriovenous malformation which has not been reported so far which can be applied especially in a resource limited setup.

## INTRODUCTION

Uterine arteriovenous malformation (AVM) is a vascular hamartoma of the myometrium characterized by the presence of the shunts between myometrial arteries and veins.^1^ The exact incidence of AVMs is not known as fewer than 100 cases have been reported in the literature though the latest imaging modalities is likely to increase the identification of such lesions more frequently.^2^ Arteriovenous malformation generally manifests through a spectrum of vaginal bleeding ranging from menorrhagia to life threatening bleeding episodes, lower abdominal pain and dyspareunia.^3^

Diagnosis of AVM can be done using noninvasive modalities such as ultrasonography (USG) with color Doppler, Computed Tomography (CT) and Magnetic resonance Imaging (MRI). Hysteroscopy can be used for detection of AVM present just beneath the endometrium. Digital subtraction angiography is the gold standard technique for diagnosing AVM.^2,4^ Arteriovenous malformations can be managed expectantly and conservatively with medical therapy such as hormonal suppression and uterotonics.^5^ Surgical management of AVM includes uterine artery embolization, hysteroscopic resection and hysterectomy.^1,6^

We hereby report a case of uterine AVM managed conventionally with Uterine Ballon Tamponade (UBT) which is generally used for managing postpartum hemorrhage. The same principle has been applied in our case with successful outcome.

## CASE REPORT

25 years P1L1 lady, a known case of abnormal uterine bleeding (AUB) secondary to uterine AVM presented to our hospital with per vaginal bleeding for one day with heavy bleeding since last four hours. Her general and systemic examination findings were unremarkable except for pallor. Pelvic examination revealed bulky uterus. AVM was diagnosed by MRI three weeks back and she was successfully managed with progesterone and discharged from the hospital with the same medication. On her follow up, USG was done which showed endometrial collection hence she was planned for Suction and Evacuation (S&E).

During the procedure, torrential bleeding was noted which was initially managed with uterotonics and antifibrinolytics. However, bleeding was not controlled and UBT placement was done. After the bleeding was controlled, the patient was managed in intensive care unit (ICU) for three days. She was transfused with three units of packed red blood cells (PRBC). Uterine balloon tamponade was removed after 3 days and no bleeding was noted. The patient was discharged after five days on progesterone in tapering dose for a month. On her regular follow up, she had no complains of per vaginal bleeding.

## DISCUSSION

Uterine arteriovenous malformation is an abnormal connection between arteries and veins without capillary bed which are prone to bleeding. It is an important differential diagnosis in reproductive age women with irregular vaginal bleeding.^7^ It can be congenital or acquired; the latter one being more common.^8^ Congenital AVM develop due to failure in embryological differentiation of primitive vascular structures leading to abnormal vascular connection. Acquired AVMs are often related to iatrogenic uterine trauma such as miscarriages, dilatation and curettage, cesarean section (CS), vaginal delivery, trophoblastic diseases, endometrial or cervical carcinoma and infections.^2^

The first reported case by Dubreil and Loubart was a cricoid aneurysm of the uterus and since then it has been referred to as arteriovenous malformation.^1,7^ A retrospective study by Calzolari S et al (2017) reported 11 cases of uterine AVM, all of which were successfully managed with operative hysteroscopy. Of these, six patients achieved a pregnancy, four carried the pregnancy till term, one was pregnant at 20 weeks and one had abortion in first trimester.^1^

A systematic review and metanalysis was carried out by Rosen A et al in 2021 where 32 studies with 121 premenopausal women with medically managed uterine AVM were identified. The overall success rate was 88%. Progestins, GnRH agonists, methotrexate, combined oral contraceptive pills, danazol were given as monotherapy or in combination. Of these, progestins and GnRH agonists were extensively studied and found more effective with less side effects. The advantage of medical therapy is that it is universally accessible, less expensive and avoids risks of embolization.^5^

Hammad R et al (2020) reported four cases of uterine AVM. Three cases were managed with selective uterine artery embolization successfully. Later on, two of them had a live birth. One case was managed medically with progesterone.^7^

Ishihara T et al published a case report (2017) in regards to placental polyp as a differential diagnosis of uterine AVM. A primipara lady on 28^th^ postpartum day following CS presented for routine checkup. Transvaginal ultrasonography revealed abnormal mass in the uterine cavity. As the patient was asymptomatic, she was followed up till 55^th^ postpartum day. Though the mass was persistent but the patient was asymptomatic with normal beta hCG value. Magnetic resonance imaging confirmed uterine AVM. Since the patient was asymptomatic no further intervention was done.^9^

There is no clear consensus regarding the best treatment option of AVM and it depends upon the age of the patient, her symptoms, desire for fertility, hemodynamic stability and site of uterine arteriovenous malformation. Expectant and medical management is reserved for stable cases. The definitive treatment is hysterectomy while selective uterine artery embolization is performed in women who wants to preserve fertility.^6^

This case emphasizes uterine AVM could be differential diagnosis of AUB. It is possible to manage the cases with acute bleeding AVM with conventional approach such as UBT or medical therapy in selected cases where fertility preservation is desired. It is more relevant in resource limited areas where uterus conserving latest technologies such as hysteroscopy and interventional radiology are not readily available.

## CONCLUSIONS

Uterine arteriovenous malformation is a rare but potentially life threatening condition as a result of torrential hemorrhage. Ultrasonography with color doppler can be the initial screening test. Uterine balloon or condom tamponade could be optional treatment modality along with hormonal therapy with progesterone.
